# Elevated neuraminidase potentiates lung inflammation through facilitating integrin β2-mediated adhesion and immune responses of neutrophils

**DOI:** 10.1016/j.jbc.2026.111311

**Published:** 2026-02-26

**Authors:** Lulu Huang, Amy Tang, Hui Zhong, Ying Liang, Bojing Shao

**Affiliations:** 1 Lab of Vascular Inflammation and Thrombosis Research, Lindsley F. Kimball Research Institute, New York Blood Center, New York, New York, USA; 2 Lab of Immune Regulation, Lindsley F. Kimball Research Institute, New York Blood Center, New York, New York, USA; 3 Lab of Stem Cell Aging and Regeneration, Lindsley F. Kimball Research Institute, New York Blood Center, New York, New York, USA

**Keywords:** sialylation, integrin, neutrophil, adhesion, and immune response

## Abstract

Acute inflammation in the lungs is a leading cause of death worldwide, with limited treatments. During the development of inflammatory lung diseases, glycosylation is altered and emerges as a potential therapeutic target. Decreased sialylation is a common change in glycosylation. Yet the role of decreased sialylation in the progression of lung inflammation is complex and remains elusive. Here, we examined the role of sialic acids on neutrophils in modulating integrin β2-regulated cell adhesion and immune responses. The removal of sialic acids increased integrin β2-mediated adhesion and immune responses of neutrophils, including cytokine release, reactive oxygen species production, and neutrophil extracellular trap formation. In addition, the removal of sialic acids did not alter the ligand-binding affinity of integrin β2 but increased the ligand-binding valency of integrin β2. In inflamed lungs, the binding of the receptor for advanced glycation end product, a ligand highly expressed on lung epithelial cells, to integrin β2 facilitated the interaction of neutrophils with lung cells, which was also enhanced after removing sialic acids. Thus, sialic acids negatively regulate the valency of integrin β2 for its ligands and the subsequent neutrophil adhesion and ligand-induced immune responses. Our study suggests a mechanism by which decreased sialylation potentiates inflammation and provides novel insights into the pathogenesis of lung inflammation.

Acute inflammation in the lungs is a pathological condition that impairs arterial oxygenation and may lead to lung failure. Despite medical innovations, the treatment of acute lung inflammation remains a challenge. Glycosylation plays a significant role in the pathogenesis of inflammatory lung diseases and may be a new target for the treatment of lung inflammation. Glycosylation, one of the common post-translational modifications of proteins and lipids, can be changed during the progression of lung inflammation. For example, because of altered expression of glycotransferases (1, 2), abnormal glycosylation is present in most inflammatory lung diseases (3, 4, 5, 6). Among the changed glycosylation in inflamed lungs, decreased sialylation is more often observed. Sialic acids play a critical role in regulating protein–protein interactions, protein conformation and stability, and cellular signaling (7). During inflammation, sialic acids in lung tissues are depleted at differing degrees by neuraminidases (NAs) (8, 9), which generally exacerbates lung inflammation (3, 4, 5, 6). However, the underlying mechanism is complicated and remains elusive.

Neutrophil infiltration is a hallmark in the progression of acute lung inflammation (10, 11). Neutrophil extravasation from the circulation into inflamed tissues is mediated by an adhesion cascade, that is, selectin-regulated leukocyte rolling and integrin-regulated leukocyte adhesion and transmigration (12). The interaction of selectins with ligands to support leukocyte rolling requires sialofucosylated glycan epitopes, including sialyl Lewis x/a and 6-sulfo sialyl Lewis x (7, 13). To mediate leukocyte adhesion, the integrin interaction with its ligands is controlled by the ligand-binding affinity and valency, that is, the number of ligand-interacting bonds (14, 15). Yet the role of glycans on integrins in modulating the interaction with ligands varies for different integrins (16, 17, 18, 19, 20, 21). Integrin αLβ2 and αMβ2 are the major integrins that regulate adhesion and transmigration of neutrophils. However, the role of glycans or sialic acids on integrin β2 in neutrophil infiltration during acute inflammation in the lungs is not well studied.

Here, we studied the role of altered sialylation in the progression of acute lung inflammation by examining how sialic acids on integrin β2 modulate adhesion and immune responses of neutrophils. Our studies showed that the level of NA in mouse lungs was elevated in response to intratracheal instillation of lipopolysaccharide (LPS), and inhibiting NA in the lungs alleviated lung inflammation. Removing sialic acids increased the valency but not the affinity of integrin β2 for its ligands and subsequently enhanced integrin β2-mediated adhesion and immune responses of neutrophils. Furthermore, in inflamed lungs, integrin β2 on neutrophils interacted with the receptor for advanced glycation end products (RAGEs) on lung epithelial cells, which were also increased by the removal of sialic acids on integrin β2. Thus, our studies revealed a mechanism by which decreased sialylation facilitates lung inflammation and provides novel insights into the pathogenesis of acute inflammation in the lungs.

## Results

### Elevated NA deteriorates lung injury during noninfectious lung inflammation

**Figure 1:**
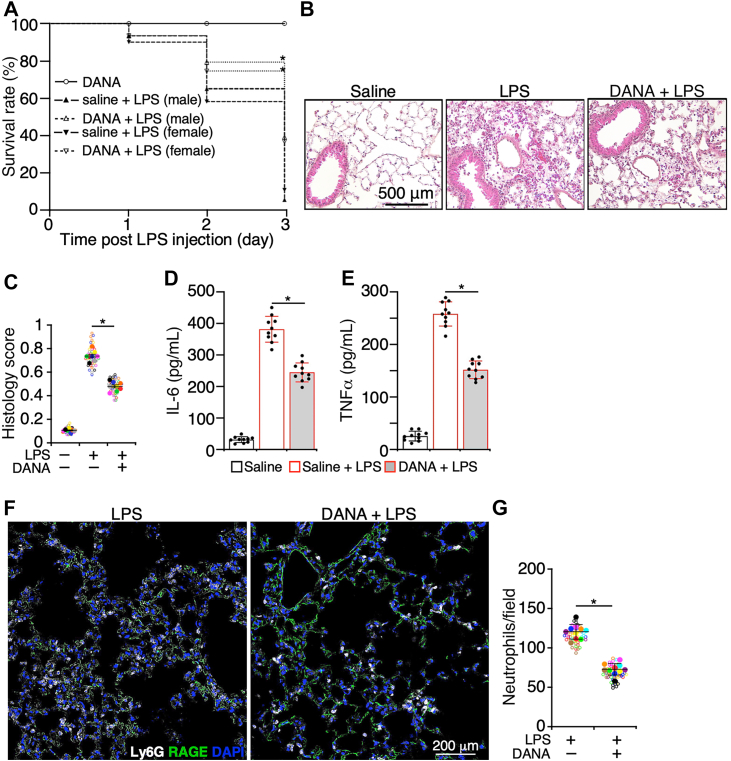
**Elevated neuraminidase deteriorates lung injury during acute lung inflammation.***A*, WT mice that were injected with LPS (10 mg/kg body weight) *via* trachea after preinjection of saline or DANA (20 μmol/mouse, i.v.), and the survival rate in 3 days post LPS injection was recorded. *B*, lung histology of WT mice at 24 h postadministration of LPS (5 mg/kg body weight) *via* trachea. Some mice were given saline or DANA (20 μmol/mouse) prior to LPS instillation. *C*, score of histology injury. *Empty circle*s in different colors represented the individual data from different pools, and *solid circles* were the average of experimental pools, shown with the *empty circles* in the same colors. The bars were the mean ± SD of the averages. (*D*) IL-6 and (*E*) TNFα in bronchoalveolar lavage fluid (BALF) of mice studied in *B* were measured. *F*, cryosections of lungs from mice studied in *B* were stained with antibodies to Ly6G (a marker for neutrophils) and RAGE (a marker for AT1 cells) and DAPI. *G*, quantification of infiltrated neutrophils in lungs studied in *B* that were challenged by LPS with or without administration of DANA. *Empty circles* in different colors represented the individual data from different pools, and *solid circles* were the average of the *empty circles* in the same colors. The bars were the mean ± SD of the averages. For the survival study, there were 10 mice in each group, and for all other studies, there were 10 mice, containing five male and five female mice in each group. Five sections of each lung sample were analyzed for histology and neutrophil recruitment. Data are mean ± SD. ∗*p* < 0.05. LPS, lipopolysaccharide. AT1, alveolar type I; DANA, 2,3-dehydro-2-deoxy-*N*-acetylneuraminic acid; DAPI, 4′,6-diamidino-2-phenylindole; IL-6, interleukin 6; RAGE, receptor for advanced end product; TNFα, tumor necrosis factor alpha.

Pathogen-derived NA exacerbates infectious lung injury. For instance, viral NA supports the secondary bacterial pneumonia (22), and bacterial NA contributes to the formation of biofilm during pulmonary infection (8). NA is also released from injured tissues (23, 24); therefore, we examined the role of NA in the progression of noninfectious lung inflammation. Acute inflammation in mouse lungs was induced through intratracheal instillation of LPS. LPS administration significantly increased the level of NA both in lung tissues (Fig. S1*A*) and bronchoalveolar lavage fluid (BALF) (Fig. S1*B*), which was prohibited by pretreatment with 2,3-dehydro-2-deoxy-*N*-acetylneuraminic acid (DANA), an inhibitor of NA. Subsequently, we tested the effect of inhibiting NA on the progression of lung inflammation. Mice were pretreated with the NA inhibitor, DANA, prior to LPS administration, which increased the survival rate of animals (Fig. 1*A*). Furthermore, LPS induced tissue damage in lungs, such as leukocyte infiltration into lungs, a mild hemorrhage, thickening of the alveolar wall, the formation of fibrous tissue plugs, and a mild collapse of airspaces, which were ameliorated by DANA (Fig. 1, *B* and *C*). Consistent with the findings of histology, administration of DANA reduced the levels of interleukin 6 (IL-6) (Fig. 1*D*) and tumor necrosis factor alpha (TNFα) (Fig. 1*E*) in BALF and the number of infiltrated neutrophils in inflamed lungs (Fig. 1, *F* and *G*). These data suggest that NA is released from injured tissues during acute noninfectious lung inflammation, and elevated NA deteriorates lung tissue injury.

### Removing sialic acids increases clustering of integrin β2 upon ligand binding

**Figure 2:**
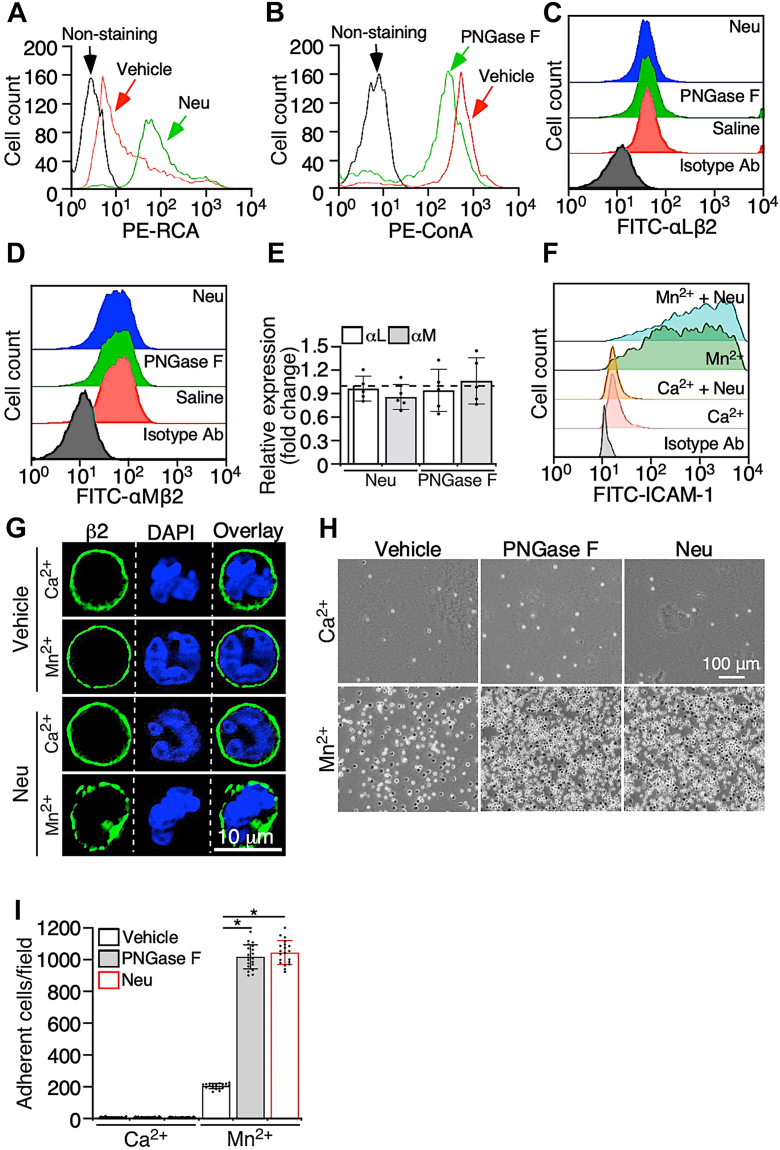
**Removing sialic acids facilitates integrin β2-mediated neutrophil adhesion through increasing the valency of integrin β2 for ligands.***A*, purified mouse neutrophils were pretreated with α2-3,6,8,9 neuraminidase (neuraminic acid [Neu]) and the vehicle control. After washing cells to remove neuraminidase, neutrophils were further incubated with the lectin Ricinus communis agglutinin. The binding of Ricinus communis agglutinin to cells was analyzed with flow cytometry. *B*, neutrophils were pretreated with PNGase F and the vehicle control, followed by incubation with lectin ConA. The binding of ConA to cells was analyzed with flow cytometry. Expression of integrin (*C*) αLβ2 and (*D*) αMβ2 on neuraminidase (Neu)- or PNGase F-treated neutrophils was measured by flow cytometry. *E*, quantitative analysis of gene expression of integrin αL and αM in neutrophils treated with the vehicle control and neuraminidase or PNGase F. The *dotted line* represents the mean expression of the control gene, GAPDH. *F*, neutrophils were pretreated with neuraminidase (Neu). After removing neuraminidase, cells were incubated with soluble ICAM-1-Fc chimera in the presence of Mn^2+^ or Ca^2+^, and binding of ICAM-1 was measured with flow cytometry. *G*, post neuraminidase (Neu) treatment, neutrophils were incubated with soluble ICAM-1. Cells were then washed and stained with antibodies to integrin β2, and the distribution of integrin β2 on the neutrophil membrane surface was imaged with confocal microscopy. *H*, neutrophils were pretreated with neuraminidase (Neu) or PNGase F. After washing cells to remove enzymes, cells were applied to ICAM-1-coated plates in the presence of Mn^2+^ or Ca^2+^, and cell adhesion was imaged. *I*, quantification of adherent cells in *H*. Images are the representatives of five independent experiments, and the data are the mean ± SD of five independent assays. ∗*p* < 0.05. The bar represents 10 μm. ICAM-1, intercellular adhesion molecule-1.

It has been shown that NA can regulate inflammation through multiple approaches (25, 26, 27, 28). Here, we aimed to examine the role of sialic acids on integrin β2 in neutrophil adhesion, as integrin β2-mediated neutrophil adhesion plays a critical role in the development of lung inflammation (10, 11). Neutrophil β2 integrins, including αLβ2 and αMβ2, are decorated with *N*-glycans (29, 30), on the termini of which sialic acid residues are associated. Sialic acids on neutrophils were removed by NA. To verify the role of sialic acids, *N*-glycans on neutrophils were removed by PNGase F, because sialic acids on integrin β2 would be dramatically reduced in the absence of *N*-glycans. The successful removal of sialic acids and *N*-glycans was confirmed by the altered binding of lectins (Fig. 2, *A* and *B*). The treatment of NA and PNGase F did not alter the expression levels of integrin αLβ2 (Fig. 2*C*) and αMβ2 (Fig. 2*D*). Furthermore, the transcription of integrin β2, including integrin αL and integrin αM, was measured. Compared with the vehicle, NA and PNGase F did not alter the gene expression of integrin αL and αM (Fig. 2*E*). Thus, removing sialic acids or *N*-glycans from integrin β2 on the membrane surface does not alter its surface expression and gene transcription in neutrophils.

Neutrophil adhesion mediated by the interaction of integrins with their ligands is controlled by the ligand-binding affinity and the number of ligand-binding bonds (*i.e*., valency) (31). We first examined whether sialic acids on integrin β2 play a role in regulating its ligand-binding affinity. The binding of soluble intercellular adhesion molecule-1 (ICAM-1) was applied to measure the affinity of integrin β2 (32). Mn^2+^ increased the binding of ICAM-1 to neutrophils (Fig. 2*F*). NA neither caused ICAM-1 binding in the presence of Ca^2+^ nor increased ICAM-1 binding after treating neutrophils with Mn^2+^ (Fig. 2*F*). Thus, sialic acids on integrin β2 may not play a role in regulating the ligand-binding affinity of integrin β2.

We then tested whether sialic acids regulate the valency of integrin β2 for its ligand. Ligand binding to integrins initiates the outside–in signaling, triggering integrin clustering on the cellular membrane, an indicator of the increased integrin valency (31). Neutrophils were incubated with ICAM-1 in the presence of Mn^2+^ or Ca^2+^, and then integrin β2 was labeled by fluorescence-conjugated antibodies (Abs). The distribution of integrin β2 on the membrane surface of neutrophils was observed. Integrin activation by Mn^2+^ did not significantly alter the distribution of integrin β2 on the neutrophil membrane surface (Fig. 2*G*). Yet, NA treatment increased the formation of integrin β2 clusters on the neutrophil membrane in the presence of Mn^2+^ (Fig. 2*G*). These data suggest that the removal of sialic acids facilitates the formation of clusters of ligand-bound integrin β2. In other words, removing sialic acids may increase the valency of integrin β2 for its ligand.

### Removing sialic acids from neutrophils increases integrin β2-mediated cell adhesion

Then we examined the role of sialic acids in regulating integrin β2-regulated neutrophil adhesion. To circumvent the defects of removing sialic acids and *N*-glycans on selectin-regulated neutrophil rolling under flow (25), we tested the role of sialic acids in integrin β2-mediated neutrophil adhesion under static conditions. Neutrophils were applied onto immobilized ICAM-1 in the presence of Mn^2+^ or Ca^2+^, and cell adhesion by the interaction of integrin β2 with ICAM-1 was measured. Divalent cation Mn^2+^ increases the ligand-binding affinity of integrins through allosterically altering the conformation of the integrin extracellular domain (33, 34), whereas Ca^2+^ is the control for Mn^2+^. The results showed that Mn^2+^ induced a significant increase in neutrophil adhesion onto ICAM-1, compared with Ca^2+^ (Fig. 2, *H* and *I*). Notably, removing sialic acids or *N*-glycans on neutrophils did not induce a spontaneous adhesion to ICAM-1 but increased neutrophil adhesion in the presence of Mn^2+^ (Fig. 2, *H* and *I*). Thus, the removal of sialic acids does not spontaneously increase the interaction of integrin β2 with ICAM-1 but facilitates this interaction upon integrin β2 activation by Mn^2+^, which leads to more neutrophil adhesion.

### Removing sialic acids on neutrophils increases integrin β2-mediated inflammatory responses of neutrophils

**Figure 3:**
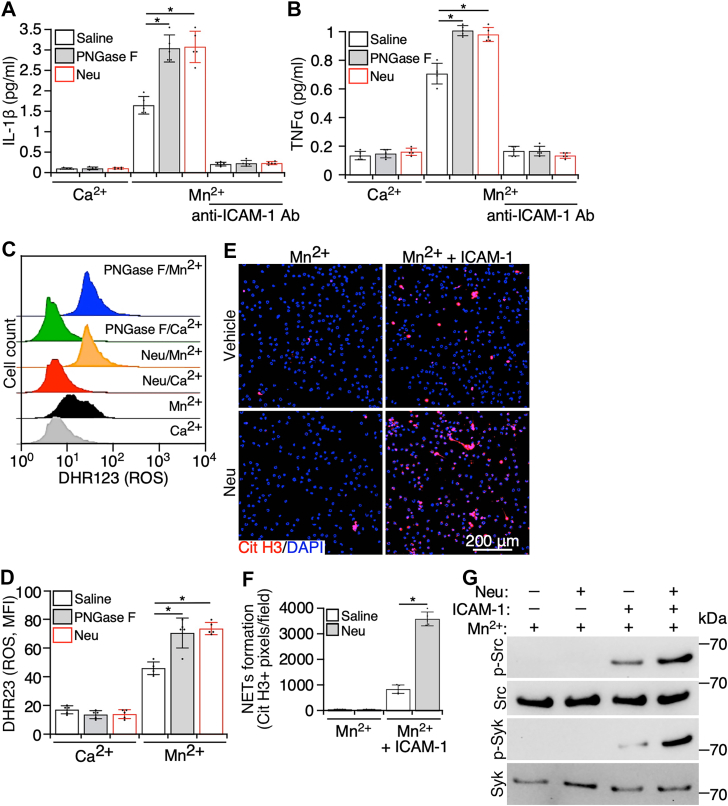
**Removing sialic acids increases integrin β2-mediated immune responses of neutrophils.** Purified mouse neutrophils were pretreated with neuraminidase (neuraminic acid [Neu]) and PNGase F to remove sialic acids and *N*-glycans, respectively. After washing cells to remove enzymes, neutrophils were applied to immobilized ICAM-1 in the presence of Mn^2+^ or Ca^2+^, and (*A*) IL-1β and (*B*) TNFα released in the supernatant were measured by ELISA. In some experiments, ICAM-1 on plates was preblocked with antibodies to ICAM-1 before the addition of cells. *C*, enzyme-treated neutrophils were also incubated with DHR123, and the signal of DHR123 in neutrophils was measured by flow cytometry. *D*, quantification of the DHR123 signal in *C*. *E*, neuraminidase (Neu)- or the vehicle-treated neutrophils were applied to polylysine- or ICAM-1-coated plates in the presence of Mn^2+^ for 2 h. Then, the adherent cells were stained with antibodies to citrullinated histone and DAPI, followed by imaging cells with confocal microscopy. *F*, quantification of citrullinated histone–positive areas in *E*. *G*, integrin β2-mediated signaling was examined with Western blot. Purified mouse neutrophils were pretreated with neuraminidase (Neu) and the vehicle control. After washing to remove neuraminidase, cells were incubated with Mn^2+^ in the presence or the absence of ICAM-1. Then, the cells were lysed, and tyrosine phosphorylation of (pan) Src and Syk was examined. Images are the representatives of five independent experiments, and the data are mean ± SD from five independent assays. ∗*p* < 0.05. DAPI, 4′,6-diamidino-2-phenylindole; ICAM-1, intercellular adhesion molecule-1; IL-1β, interleukin 1 beta; TNFα, tumor necrosis factor alpha.

Ligand binding to integrin β2 is able to induce immune responses in neutrophils (35). Thus, we examined the effect of sialic acids on integrin β2-regulated immune responses of neutrophils. First, neutrophils were incubated with ICAM-1 in the presence of Mn^2+^ or Ca^2+^, and the inflammatory cytokines released into the supernatant were measured. In the presence of Ca^2+^, IL-1β and TNFα produced by neutrophils were at the basal levels, and Mn^2+^ increased the cytokine production in neutrophils (Fig. 3, *A* and *B*). The cytokine production was further elevated from NA- and PNGase F-treated neutrophils. In addition, the cytokine production could be abolished by Abs against ICAM-1 (Fig. 3, *A* and *B*). These data suggest that this cellular event is triggered by the interaction of integrin β2 with its ligand ICAM-1 after Mn^2+^ increases the ligand-binding affinity of integrin β2.

During inflammation, neutrophils produce reactive oxygen species (ROS) to damage tissues and kill pathogens. Ligand binding to integrin β2 on neutrophils can induce ROS production (35). In the presence of ICAM-1, Mn^2+^ stimulated neutrophils to produce ROS, which was detected by DHR123 (Fig. 3, *C* and *D*). The removal of sialic acids or *N*-glycans *per se* did not alter ROS production in neutrophils, but it potentiated Mn^2+^-triggered ROS production (Fig. 3, *C* and *D*).

Neutrophil extracellular traps (NETs) are neutrophil-specific web-like structures formed during inflammation, which are composed of neutrophil DNA, histones, and cytosolic and granule proteins (36). As a double-edged sword, NETs can damage tissues, induce thrombosis, and eliminate invaded pathogens as well (36). Here, we examined the role of sialic acids on neutrophil integrin β2 in the NET formation. Mn^2+^ or NA alone did not initiate the NET formation by neutrophils (Fig. 3, *E* and *F*). However, Mn^2+^ caused the NET formation by neutrophils in the presence of ICAM-1, which was further enhanced by NA treatment (Fig. 3, *E* and *F*). The results suggest that the interaction of integrin β2 with ICAM-1 initiates the NET formation, and this process is augmented by removing sialic acids on integrin β2.

Furthermore, we examined the effects of sialic acids on integrin signaling. Integrins mediate the signaling upon ligand binding, such as activation of Src and Syk (37). Activation of Src (phosphorylation of tyrosine 416) and Syk (phosphorylation of tyrosine 519/520) in neutrophils incubated with Mn^2+^ in the presence or the absence of ICAM-1 was detected with Western blot. There was no significant signaling transduction in Mn^2+^-treated cells, whereas activation of integrins to the high-affinity state by Mn^2+^ allowed ICAM-1 to induce activation of Src and Syk (Fig. 3*G*). Compared with the vehicle control, removing sialic acids by NA enhanced ICAM-1-induced activation of Src and Syk (Fig. 3*G*). Thus, the enhanced valency may increase the integrin signaling after ligand engagement.

Taken together, these data indicate that the removal of sialic acids increases ligand binding to activated integrin β2. This alteration, in turn, elevates the production of inflammatory cytokines, ROS, and NETs from neutrophils that may facilitate inflammation.

### Removing sialic acids enhances RAGE binding to integrin β2 on neutrophils

**Figure 4:**
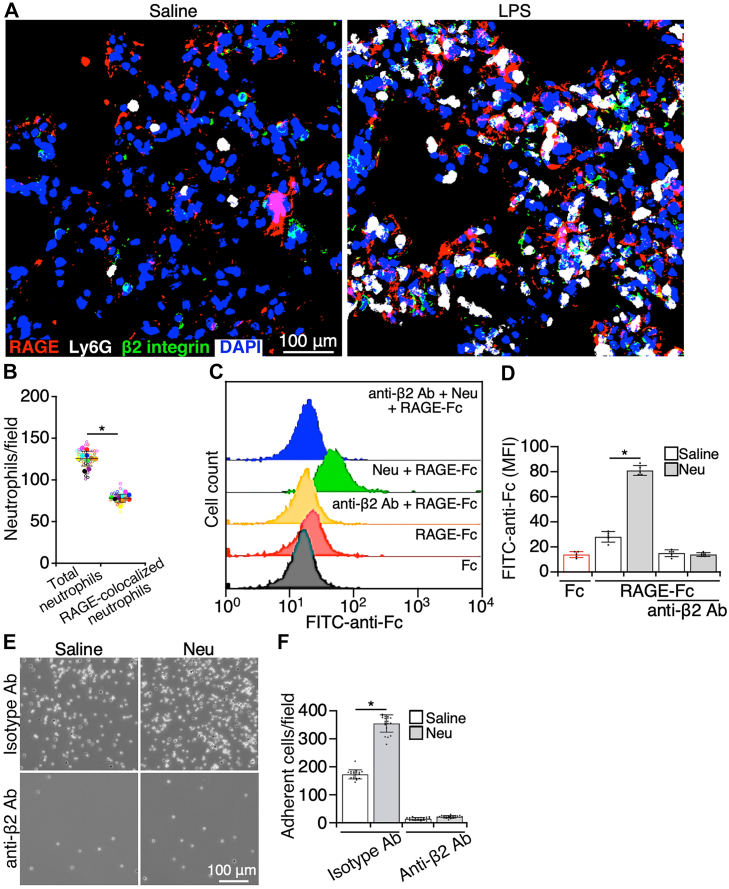
**Removing sialic acids increased the binding of RAGE to integrin β2 on neutrophils.***A*, cryosections of lungs from mice treated with saline or LPS (10 mg/kg body weight) for 24 h were stained with antibodies to RAGE (a marker of AT1 cells), Ly6G (a marker of neutrophils), and integrin β2 and DAPI, followed by imaging with confocal microscopy. There were 10 mice, containing five male and five female mice in each group. *B*, quantification of the interaction of neutrophils with AT1 cells. Infiltrated neutrophils in the lungs of LPS-challenged mice, shown in *A*, were counted and shown as total infiltrated neutrophils and neutrophils that were colocalized with RAGE on AT1 cells. Five sections of each lung sample were analyzed. *Empty circles* in different colors represented the individual data from different pools, and *solid circles* were the average of experimental pools shown with the *empty circles* in the same colors. The bars were the mean ± SD of the averages. *C*, neutrophils were treated with neuraminidase (Neu). After washing cells to remove the enzyme, neutrophils were incubated with recombinant RAGE, and binding of RAGE to neutrophils was measured by flow cytometry. In some experiments, neutrophils were treated with antibodies to integrin αMβ2 (anti-β2 antibody [Ab]) before the addition of RAGE. *D*, quantification of RAGE binding in *C*. *E*, neutrophils were treated with saline or neuraminidase (Neu), followed by applying cells to RAGE-coated plates and imaging adherent cells. In some experiments, neutrophils were treated with antibodies to integrin αMβ2 (anti-β2 Ab) or isotype controls (isotype Ab) before being added the plates. *F*, quantification of adherent cells in *E*. Images are the representatives of five independent experiments, and the data are the mean ± SD of five independent assays. ∗*p* < 0.05. AT1, alveolar type I; DAPI, 4′,6-diamidino-2-phenylindole; LPS, lipopolysaccharide; Neu, neuraminic acid; RAGE, receptor for advanced glycation end product.

RAGE is a surface protein highly expressed on alveolar type I (AT1) epithelial cells in the lungs (38). RAGE is a ligand for αMβ2 (39); however, the role of RAGE binding to neutrophil integrin β2 in the development of lung inflammation remains to be investigated. First, we examined the interaction of integrin β2 on neutrophils with RAGE on AT1 cells in inflamed lungs. In cryosections of inflamed lung tissues, the immunofluorescence staining of integrin αMβ2, neutrophils, and RAGE colocalized with each other, suggesting that integrin αMβ2 on infiltrated neutrophils could interact with RAGE on AT1 cells in inflamed lungs (Fig. 4, *A* and *B*).

We then proceeded to examine the role of sialic acids on integrin β2 in regulating the interaction of RAGE with neutrophil integrin αMβ2. Incubation of neutrophils with soluble RAGE showed a minimal binding of RAGE to neutrophils (Fig. 4, *C* and *D*). However, removing sialic acids from neutrophils by NA resulted in a significant increase in RAGE binding. The increased binding was prohibited by Abs against integrin αMβ2 (Fig. 4, *C* and *D*). Similarly, neutrophils were capable of adhering to immobilized RAGE, which was increased by removing sialic acids on neutrophils and inhibited by Abs to integrin β2 (Fig. 4, *E* and *F*). Furthermore, the interaction of integrin β2 with RAGE in mediating neutrophil adhesion on AT1 cells was examined. Purified neutrophils were labeled with fluorescence dye and applied to cultured primary AT1, and cell adhesion was imaged (Fig. S2). Neutrophils were able to adhere onto AT1 cells, and cell adhesion was decreased by Abs to integrin β2 and RAGE (Fig. S2). Thus, integrin β2 and RAGE mediate neutrophil interaction with AT1 cells, and the removal of sialic acids on integrin αMβ2 enhances RAGE binding to neutrophils, leading to an increase in the interaction of neutrophils with lung epithelial cells during inflammation.

## Discussion

**Figure 5:**
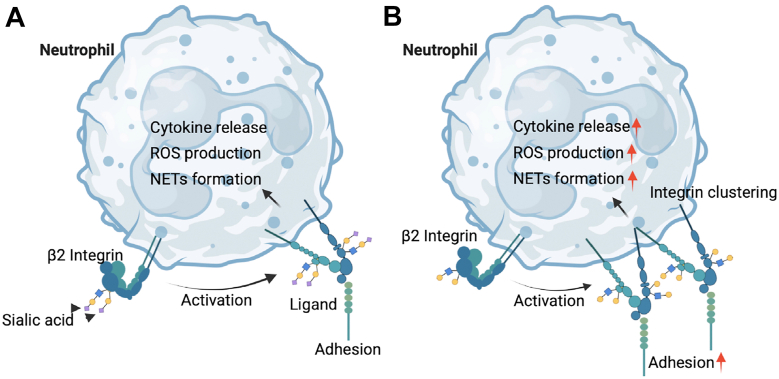
**Graphical summary.***A*, interaction of integrin β2 with its ligands mediates adhesion and immune responses of neutrophils to regulate the progression of inflammation. *B*, the removal of sialic acids on integrin β2 increases the valence of integrin β2 for its ligands, which facilitates adhesion and immune responses of neutrophils, leading to enhanced inflammation.

Neutrophil infiltration exerts a substantial influence on the severity of acute lung inflammation (10, 11). Integrin β2-regulated neutrophil adhesion is a key step for the neutrophil influx into inflamed lungs. Integrin β2 controls neutrophil adhesion by increasing the conformation-associated affinity for ligands and/or the number of integrin–ligand bonds (valency) (14, 15). Upon ligand binding, integrin β2 also triggers immune responses in neutrophils, thereby contributing to tissue damage and pathogen elimination during inflammation. Glycosylation, particularly sialylation, is frequently altered during inflammatory lung diseases (9, 23). Two major types of β2 integrins on neutrophils, composed of αL/M and β2 subunits, are decorated with sialic acids. Yet, how sialic acids on integrin β2 regulate neutrophil trafficking and functions in inflammation remains elusive. Our studies suggest that removing sialic acids enhances integrin β2-mediated adhesion and immune responses of neutrophils. The removal of sialic acids also increases the binding of RAGE to integrin β2 (αMβ2), which may enhance the interaction of neutrophils with lung epithelial cells. Our studies further suggest that removing sialic acids facilitates clustering of integrin β2, indicating a higher valency, but does not alter the ligand-binding affinity of integrin β2 (Fig. 5). Thus, our studies reveal that the increased valency of neutrophil integrin β2 for its ligands may be an approach through which the reduced sialylation contributes to the progression of lung inflammation.

Glycosylation regulates numerous vital cellular processes, such as immune cell trafficking, cellular signaling, and gene transcription (7). Importantly, glycosylation can be altered under diverse pathophysiological conditions (40). For example, inflammation alters the expression of glycosyltransferases and the activity of enzymes that modify glycans (41, 42, 43, 44). Abnormal protein glycosylation has been observed in almost all inflammatory lung diseases (45), among which the alteration of sialic acids is often observed. During infectious inflammation, the level of NA, which digests sialic acids, in lung tissues is increased (23, 24). NA during lung inflammation is generated by a variety of organisms, including viruses (9), bacteria (8), lung epithelial cells, and the blood endothelial cells (23, 24). Intratracheal instillation of NA potentiates lung inflammation in LPS-treated mice (46). The inhibition of NA protects human patients with influenza-induced pneumonia (47) and reduces airway hypersensitivity in mice with asthma (48, 49). However, removing sialic acids reduces inflammation by prohibiting myeloid cell infiltration into inflamed tissues (50). Thus, how sialic acids contribute to the inflammation development should be studied under distinct pathological conditions.

We explore how sialic acids contribute to lung inflammatory injury by focusing on their roles in regulating the integrin β2–ligand interaction. Deleting *N*-acetylglucosaminyltransferase V, which catalyzes the addition of β1,6-GlcNAc branching of *N*-glycans, increases cell adhesion *via* integrin α5β1 (51). Conversely, expressing *N-*acetylglucosaminyltransferase III, which catalyzes the addition of the bisecting GlcNAc branch on *N*-glycans, decreases integrin α5β1-mediated cell adhesion to fibronectin (52). Mutation of the *N*-glycan site, N-320, on integrin β3 increases the ligand-binding affinity of integrin αIIbβ3 but not αvβ3 (18). Thus, the role of glycosylation in regulating the integrin–ligand interaction varies among different integrins. Our studies show that removing sialic acids does not cause spontaneous cell adhesion and alter the binding of soluble ICAM-1, which indicates that sialic acids do not play a role in regulating the ligand-binding affinity of integrin β2. Yet, the removal of sialic acids increases the cluster formation of integrin β2 in the presence of both Mn^2+^ and ICAM-1, suggesting that upon ligand binding, sialic acids negatively regulate the density of integrin β2 at the adhesive surface, that is, valency. This change consequently enhances the integrin-mediated neutrophil adhesion and immune responses. Our findings are supported by previous studies showing that the intratracheal instillation of NA deteriorates lung inflammation (46). Yet, our studies do seem not consistent with studies that show blocking *N*-glycans on integrin αMβ2 with lectins reduces neutrophil transmigration *in vitro* (53). However, this study also shows that lectins increase neutrophil adhesion by integrin β2. Thus, the discrepancy may come from the effect of lectins on endothelial cells during neutrophil extravasation. Therefore, sialic acids on neutrophil integrin β2 may protect lungs from inflammatory injury.

To comprehend the significance of our studies, it is critical to elucidate the pathological scenario that our findings may be applied for. Lung inflammation can be caused by insults that primarily affect lung structures, such as pulmonary infection of bacteria and viruses, and noxious stimuli from outside the lungs, such as sepsis (54, 55). Injury from direct insults to lung structures is primarily localized within lung tissues, whereas the indirect injury of lung structures mainly shows pathological changes within the circulation (56, 57). Given the essential role of sialic acids for selectins in recruiting neutrophils from the circulation into inflamed tissues, our findings offer a potential explanation for the progression of lung inflammation caused by direct injury. To be noted, ICAM-1 is decorated with *N*-glycans in high mannose/hybrid and complex (specifically α2,6-sialylated) forms, in which hypoglycosylated ICAM-1 binds to integrins with a higher affinity (58, 59, 60). Similarly, removing sialic acids on vascular cell adhesion molecule-1 facilitates leukocyte adhesion (61). Thus, the increased level of NA may facilitate adhesion and immune responses of neutrophils through lowering sialic acids on ligands of integrin β2 as well.

Our study also reveals the physiological significance of the interaction between RAGE and αMβ2 in the progression of inflammation. RAGE binding to integrin β2 mediates the interaction of neutrophils with lung cells, whereas elevated NA may enhance inflammation through increasing the interaction of integrin β2 with RAGE. Interestingly, RAGE binding does not rely on the presence of Mn^2+^, which indicates that RAGE binding is independent of the affinity of integrins. Thus, the valency of integrin αMβ2 is critical in regulating its interaction with RAGE.

Taken together, our findings on the role of sialic acids on integrin β2 in the regulation of adhesion and immune response of neutrophils provide new insights into the pathogenesis of inflammatory lung disease. Further analysis of the alteration of sialylation and/or glycosylation on recruited immune cells and lung cells during acute inflammation may benefit the development of novel therapeutics for the disease. To be noted, a limitation of our study is the application of purified neutrophils from bone marrow. Those neutrophils are not exposed to the inflammatory milieu of the tissue. Thus, the role of sialylation and glycosylation in regulating the integrin-mediated neutrophil responses is more complicated in the inflammatory environment.

## Experimental procedures

### Mice

All mouse experiments were performed in compliance with protocols approved by the Institutional Animal Care and Use Committee of the New York Blood Center. WT mice (C57BL/6J) were purchased from The Jackson Laboratory (#000664). All mice were 8 to 12 weeks old at the time of the study.

### Mouse model of acute lung inflammation

Male and female mice of 8 to 12 weeks old were used in the study. Mice were anesthetized with ketamine (100 mg/kg) and xylazine (10 mg/kg) *via* intraperitoneal injection. Then, the mice were given LPS (10 mg/kg body weight, Sigma, from *Escherichia coli* O111:B4) with a mixture of LPS (10 mg/ml in PBS) and PBS in a total volume of 60 μl through intratracheal instillation. Mice were administered PBS alone as the control. The survival rate of mice within 72 h postinjection of LPS or PBS was analyzed. In some study groups, 200 μl of DANA (100 mM, Sigma) was given to mice through the tail vein 30 min before LPS administration.

### Collection of BALF and lungs

BALF was collected with three serial instillations of 0.8 ml of sterile PBS *via* the tracheostomy at the indicated time points post LPS injection. BALF samples were centrifuged at 3000*g* for 10 min at 4 °C to remove cells, and the supernatant of BALF was aliquoted and frozen at −80 °C. To collect lungs, mice at 24 h after LPS or saline injection were perfused with 10 ml of PBS and 10 ml of 4% paraformaldehyde (PFA) (Fisher Scientific) sequentially. Then lungs were collected and fixed in 4% PFA and 10% neutral buffered formalin (Fisher Scientific) for preparing cryosections and paraffin sections, respectively.

### Histological analysis

After fixation in 10% neutral buffered formalin overnight, lungs were embedded in paraffin and sectioned at 5 μm thickness. H&E staining was performed for the evaluation of tissue morphological changes. Slides were imaged using a Nikon Eclipse E600 microscope. The histological score of lung injury was calculated as in previous publications (62, 63). Briefly, injury score = ([20 × score of neutrophils in the alveolar space] + [14 × score of neutrophils in the interstitial space] + [7 × score of hyaline membranes] + [7 × score of proteinaceous debris filling the airspaces] + [2 × score of alveolar septal thickening])/(Number of fields × 100). Five sections of each sample were analyzed for histology.

### Measurement of NA activity

The activity of NA was measured with the NA assay kit (Sigma) following the manufacturer’s instructions. Briefly, 70 μl of the NA assay buffer, 10 μl of BALF or lysates of lung tissues, and 10 μl of Milli-Q water were added to each well of the 96-well plate. After mixing for 1 min, 10 μl of the NA fluorescent substrate was added and incubated for 30 min at 37 °C. Then, the NA activity was measured using a microplate reader (BioTek, FLx800) with the excitation wavelength at 322 nm and the emission wavelength at 450 nm.

### Immunofluorescence staining

Lungs fixed in 4% PFA overnight were dehydrated with 20% sucrose, followed by embedding in optimal cutting temperature compound (Tissue-Tek, Sakura Finetek). Tissue blocks were sectioned at 20 μm thickness and stained with primary Abs against CD18 (integrin β2, BioLegend, M18/2), Ly6G (BioLegend, 1A8), and RAGE (BioLegend, EPR21171), as well as 4′,6-diamidino-2-phenylindole (ThermoFisher). Slides were imaged using a Nikon C2 confocal microscope.

### Measurement of cytokines

Purified neutrophils were treated with a2-3,6,8,9 NA (1 μg/ml, New England Biolabs), PNGase F (2 μg/ml, New England Biolabs), or the vehicle control for 30 min at 37 °C. Then cells were washed one time to remove enzymes and resuspended in Hank’s balanced salt solution (HBSS) within 2 × 10^6^ cells/ml. ICAM-1 (1 μg/ml, R&D) was precoated in 24-well plates overnight at 4 °C, and neutrophils were added to ICAM-1-coated plates in the presence of Ca^2+^ (1 mM) or Mn^2+^ (2 mM) at 37 °C for 2 h, and the supernatant was collected. The levels of TNFα and IL-1β in the supernatant were measured using ELISA according to the manufacturer’s instructions (BioLegend). For the levels of IL-6 and TNFα in BALF, BALF was collected from mice at 24 h postadministration of LPS and measured using ELISA (BioLegend). Briefly, capture Abs against corresponding cytokines were diluted and coated on a 96-well microtiter plate (Immulon 4HBX; Thermo Fisher Scientific) overnight at 4 °C. After blocking, standards and samples were added to the plate and incubated for 2 h at room temperature. Then, the plate was washed, and diluted detection Abs were added for 1 h of incubation at room temperature, followed by another 1 h incubation with avidin-horseradish peroxidase at room temperature. After washing, 3,3′,5,5′-tetramethylbenzidine substrate was added, and the reaction was stopped by sulfuric acid. Finally, absorbance was read at 450 nm using a FLUOstar Omega microplate reader, and the absorbance at 570 nm was subtracted.

### Static adhesion of neutrophils

Neutrophils were purified from mouse bone marrow as described previously (64). Neutrophils were treated with NA (1 μg/ml), PNGase F (2 μg/ml), or the vehicle control for 30 min at 37 °C, followed by washing with HBSS without Ca^2+^ and Mg^2+^. Then, the neutrophils were resuspended with HBSS without Ca^2+^ and Mg^2+^ in a concentration of 0.5 × 10^6^/ml in the presence of CaCl_2_ (1 mM) or MnCl_2_ (2 mM) and seeded onto immobilized ICAM-1 for 30 min at 37 °C. After gently removing nonadherent cells by washing, adherent cells were fixed with 2% PFA at room temperature for 10 min. Then, the adherent cells were imaged with microscopy. Adherent cells in 5 to 10 fields on the coated ICAM-1 were imaged, and experiments were repeated five times.

In some experimental groups, pretreated neutrophils were seeded onto coated RAGE (R&D) in the presence of Abs to integrin β2 or the isotype Abs for 30 min at 37 °C, and adherent cells were analyzed.

For adhesion of neutrophils on primary epithelial AT1 cells, mouse AT1 cells were cultured as previously reported by our laboratory (65). Purified neutrophils (2 × 10^5^) were labeled with CellTracker Green 5-chloromethylfluorescein diacetate (Thermo Fisher) and applied to confluent AT1 cells at 37 °C for 1 h. After removing nonadherent cells, the cells were fixed with 2% PFA and imaged. In some groups, Abs (10 μg/ml) to integrin β2 and RAGE were used to treat neutrophils and AT1 cells before the adhesion assay, respectively. Adherent neutrophils were quantified by counting the adherent cells in five fields of each assay.

### Flow cytometry

Neutrophils were purified from mouse bone marrow as described previously (64). Briefly, bone marrow leukocytes were incubated with a cocktail containing 1 μg/ml of biotinylated monoclonal Abs to CD5, CD45R, CD49b, C-kit/CD117, F4/80, TER119, CD115, and CD41 on ice. Washed cells were incubated with streptavidin magnetic beads (Miltenyi Biotec) and separated on an EasySep Magnet (Stemcell Technologies). Negatively selected neutrophils were treated with NA (1 μg/ml), PNGase F (2 μg/ml), or the vehicle control for 30 min at 37 °C, followed by washing with HBSS without Ca^2+^ and Mg^2+^ to remove enzymes in the reactions. Cleavage of *N*-glycans and sialic acids on neutrophils was analyzed by the binding of ConA (ThermoFisher) and RCA1 (Vector Lab) with flow cytometry, respectively. The expression of integrins αLβ2 (BioLegend, M17/4) and αMβ2 (BioLegend, M1/70) was also examined by flow cytometry.

To measure production of ROS in neutrophils, cells after treatment of PNGase F or NA were further incubated with Ca^2+^ (1 mM) and Mn^2+^ (2 mM) at 37 °C for 15 min. After that, cells were further incubated with DHR123 (10 μg/ml, ThermoFisher) for another 15 min. DHR123 inside the cells was detected by flow cytometry.

The binding of RAGE to neutrophils was also measured by flow cytometry. NA- and vehicle-treated neutrophils were incubated with recombinant RAGE-Human Fc (10 μg/ml) for 30 min at 37 °C. After washing cells to remove the unbound RAGE, cells were incubated with FITC-conjugated anti-human Fc (ThermoFisher) to analyze RAGE binding to neutrophils. In some groups, cells were pretreated with Abs to integrin β2 before the addition of RAGE.

### Western blot

Purified neutrophils were treated with the vehicle control or NA (1 μg/ml) for 30 min at 37 °C. After removing NA, neutrophils (2 × 10^6^) were then incubated with Mn^2+^ for 15 min at 37 °C, and then ICAM-1 (2 μg/ml) was added to cells for another 15 min. Cells were lysed on ice with 1% Triton X-100. Total and activated Src (pTyr416) and Syk (pTyr519/520) were probed by Western blots. Abs for Src and Syk were from Cell Signaling.

### Real-time quantitative PCR

Purified neutrophils were treated with a2-3,6,8,9 NA (1 μg/ml), PNGase F (2 μg/ml), or the vehicle control for 30 min at 37 °C. Then cells were washed one time to remove enzymes. Total RNA was extracted from neutrophils using the RNeasy Kit (Qiagen) as instructed by the manufacturer. Reverse transcription was carried out using the Omniscript RT Kit as instructed by the manufacturer. The derived complementary DNA was subject to RT–quantitative PCR (qPCR) analysis by mixing with primers and SYBR Green Master Mix (Bio-Rad). The primer sequences were as follows: Gapdh: forward 5′-TCTCCACACCTATGGTGCAA-3′ and reverse 5′-CAAGAAACAGGGGAGCTGAG-3′; Itgal: forward 5′-CATGTTCTTGCTGACCAATACCTT-3′ and reverse 5′-CTTGCCAATCCCGATGATGTA-3′; and Itgam: forward: 5′-ATCTTGAGGAACCGTGTCCAAA-3′ and reverse 5′-GGATCAAGTTGGTATTGCCATCA-3′. RT–qPCR data acquisition and analysis was performed using the Bio-Rad CFX Manager 3.0 software. The △Ct method was used for the comparison of relative expression, whereas the △△Ct method was used to compare the relative normalized expression.

### Measurement of NETs

Detection of NET formation was described in the previous study (66). Briefly, neutrophils were treated with NA (1 μg/ml) and the vehicle control at 37 °C for 30 min. After washing cells to remove NA, neutrophils were suspended in Dulbecco’s modified Eagle's medium–Ham F12 medium containing 15 mM Hepes. These cells were incubated in chamber slides (Lab-Tek, #1774902), precoated with ICAM-1 or polylysine (Sigma) in the presence of Mn^2+^ (2 mM) for 2 h at 37 °C/5% CO_2_. Then cells were fixed/permeabilized with the BD fixation permeabilization kit (#554714). They were then incubated with 0.5 μg/ml rabbit anti-mouse citrullinated histone (ThermoFisher) for 1 h at room temperature, washed, and stained with PE-conjugated anti-rabbit IgG. Finally, DNA was stained with 1 μM 4′,6-diamidino-2-phenylindole for 10 min. The chambers were removed, and the coverslips were put on after adding fluorescence mounting medium (Dako, #S3023). Fluorescent images were visualized with an Axiovert 200 fluorescence microscope (Zeiss) using a 20X objective. Images were captured using NIS-Elements software (Nikon). For each experiment, neutrophils containing citrullinated histones were identified as those that exhibited fluorescence above the baseline level of neutrophils incubated with rabbit IgG control. Cells releasing NETs were positive for both extracellular citrullinated histone and DNA. Six fields were randomly chosen per well for image acquisition, and the citrullinated histone–positive area was quantified per field.

### Quantification and statistical analysis

All data are presented as mean ± SD. For comparisons between two groups, a two-tailed, unpaired Student’s *t* test was used when the normality assumption was met using the Shapiro–Wilk test. If the normality assumption was not met, the Mann–Whitney test was used to analyze the difference between two groups. For qPCR studies, a one-sample *t* test was performed to check the significance of analyzed genes with the hypothetical value of 1, which was acquired by the normalized house-keeping gene. Kaplan–Meier survival curves between different groups were compared using the log-rank test. *p* < 0.05 was considered statistically significant. Data were analyzed using GraphPad Prism 10 (GraphPad Software, Inc).

## Data availability

All data reported in this article will be shared by the lead contact upon request. This article does not report original code. Any additional information required to reanalyze the data reported in this article is available from the lead contact upon request.

## Supporting information

This article contains supporting information.

## Conflict of interest

The authors declare that they have no conflicts of interest with the contents of this article.
