# Supplementary material for: Elevated neuraminidase potentiates lung inflammation through facilitating integrin β2-mediated adhesion and immune responses of neutrophils
Source: J Biol Chem. 2026 Feb 26;302(4):111311. doi: 10.1016/j.jbc.2026.111311 (PMC13022655; doi:10.1016/j.jbc.2026.111311)
Supplement: Supplemental Materials [file mmc1.docx]

**
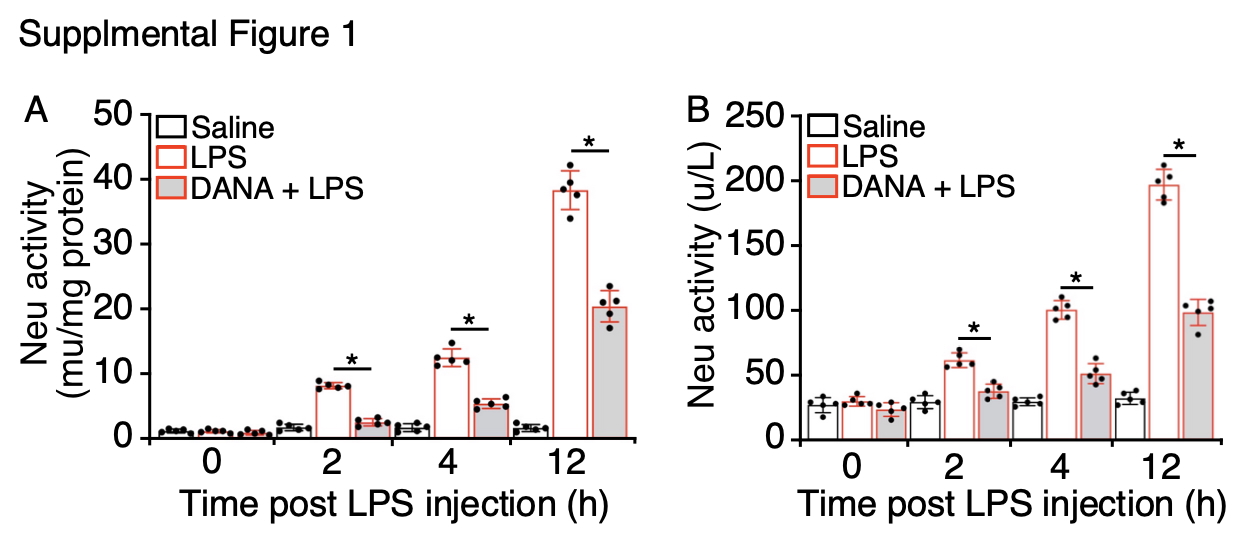
**

**Supplemental Figure 1.** DANA treatment decreases the activity of neuraminidase in lungs.

WT mice were given LPS (5mg/kg bw) via trachea for 24 h with or without of pre-injection of DANA via tail vein, neuraminidase activity in **A.** lung tissues and **B.** BALF was measured from mice at different time-points post LPS challenge. There were 5 mice in each group. Data are mean ± sd. *, *p* < 0.05.


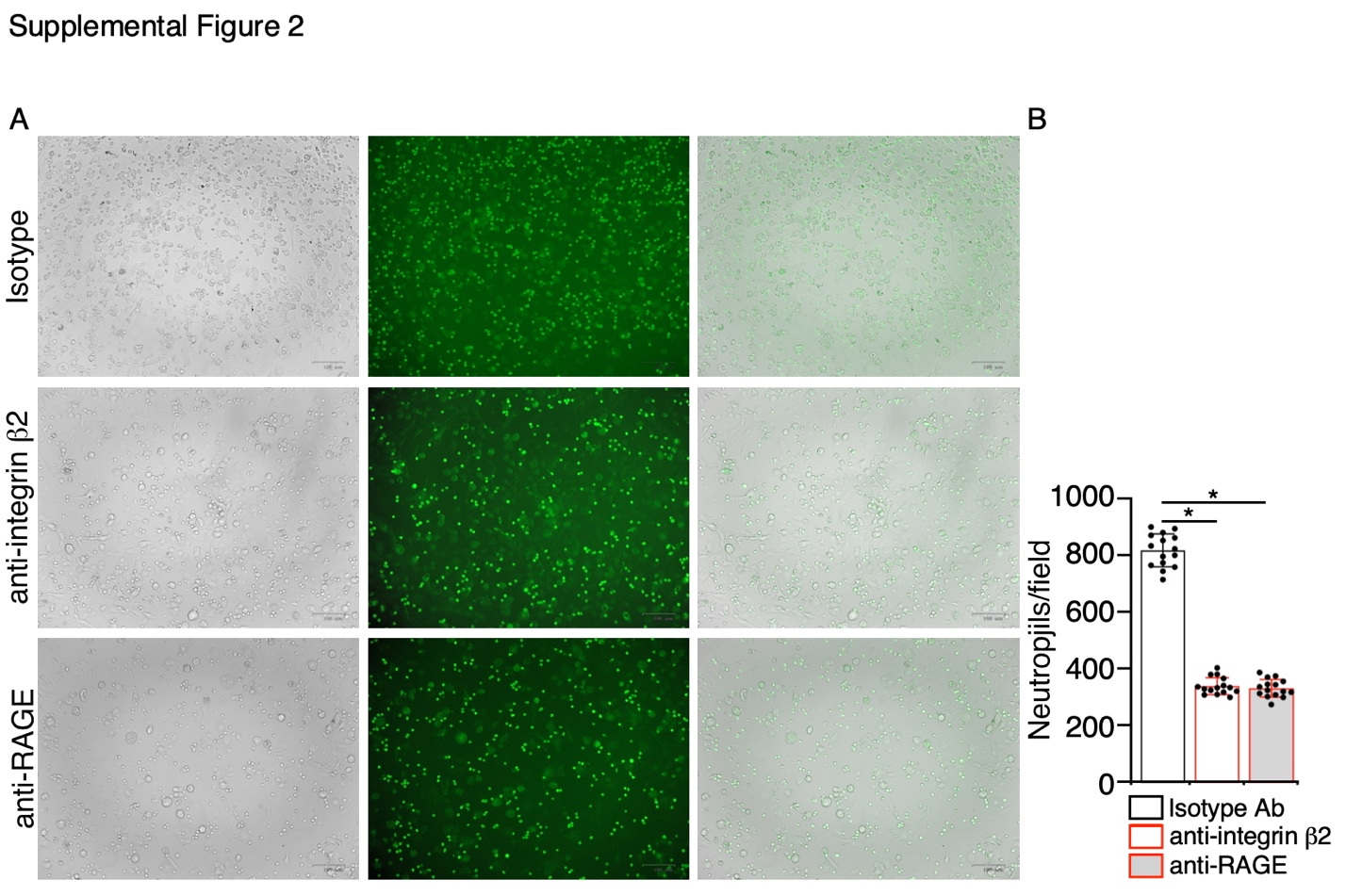
**Supplemental Figure 2.** Interaction of integrin β2 with RAGE mediates neutrophil adhesion onto AT1 cells.

Primary AT1 cells were isolated from WT mice and cultured in vitro. Then purified mouse neutrophils were labeled with green fluorescence dye and applied onto cultured AT1 cells for 1 h in the presence of isotype antibodies and blocking antibodies to integrin β2 and RAGE. After removing non-adherent cells, **A.** cells were imaged and **B.** Quantified. Images were representative of three independent assays, and cell number was collected by analyzing five field of each assay from three independent experiments. Data are mean ± sd. *, *p* < 0.05.
